# Differential Diagnosis of Vertigo in the Emergency Department: A Prospective Validation Study of the STANDING Algorithm

**DOI:** 10.3389/fneur.2017.00590

**Published:** 2017-11-07

**Authors:** Simone Vanni, Rudi Pecci, Jonathan A. Edlow, Peiman Nazerian, Rossana Santimone, Giuseppe Pepe, Marco Moretti, Andrea Pavellini, Cosimo Caviglioli, Claudia Casula, Sofia Bigiarini, Paolo Vannucchi, Stefano Grifoni

**Affiliations:** ^1^Department of Emergency Medicine, Ospedale Versilia, Azienda USL Toscana Nord Ovest, Firenze, Italy; ^2^Audiology Clinic, Azienda Ospedaliero-Universitaria Careggi, Firenze, Italy; ^3^Department of Emergency Medicine, BIDMC, Boston, MA, United States; ^4^Neuroradiology Unit, Azienda Ospedaliero-Universitaria Careggi, Firenze, Italy

**Keywords:** vertigo, unsteadiness, stroke, diagnosis, nystagmus

## Abstract

**Objective:**

We investigated the reliability and accuracy of a bedside diagnostic algorithm for patients presenting with vertigo/unsteadiness to the emergency department.

**Methods:**

We enrolled consecutive adult patients presenting with vertigo/unsteadiness at a tertiary hospital. STANDING, the acronym for the four-step algorithm we have previously described, based on nystagmus observation and well-known diagnostic maneuvers includes (1) the discrimination between **S**pon**TA**neous and positional nystagmus, (2) the evaluation of the **N**ystagmus **D**irection, (3) the head **I**mpulse test, and (4) the evaluation of equilibrium (sta**N**din**G**). Reliability of each step was analyzed by Fleiss’ *K* calculation. The reference standard (central vertigo) was a composite of brain disease including stroke, demyelinating disease, neoplasm, or other brain disease diagnosed by initial imaging or during 3-month follow-up.

**Results:**

Three hundred and fifty-two patients were included. The incidence of central vertigo was 11.4% [95% confidence interval (CI) 8.2–15.2%]. The leading cause was ischemic stroke (70%). The STANDING showed a good reliability (overall Fleiss *K* 0.83), the second step showing the highest (0.95), and the third step the lowest (0.74) agreement. The overall accuracy of the algorithm was 88% (95% CI 85–88%), showing high sensitivity (95%, 95% CI 83–99%) and specificity (87%, 95% CI 85–87%), very high-negative predictive value (99%, 95% CI 97–100%), and a positive predictive value of 48% (95% CI 41–50%) for central vertigo.

**Conclusion:**

Using the STANDING algorithm, non-sub-specialists achieved good reliability and high accuracy in excluding stroke and other threatening causes of vertigo/unsteadiness.

## Introduction

Vertigo is the illusory sense of movement, while unsteadiness is the feeling of being unstable while seated, standing, or walking (1). These two common symptoms (2) are common reasons for presentation to physicians’ offices, absence from work, and disability (3), and are responsible of 1–3% of presentations to the emergency department (ED) (4). Most cases are caused by benign diseases of the inner ear (5, 6). However, these symptoms may also be an indicator of more serious central nervous system diseases, including stroke, neoplasm, or demyelinating disease (7–9). The incidence of cerebrovascular disease in patients presenting to the ED with these complaints ranges from 3 to 5% (10, 11). Among ED patients, vertigo is the most common symptom associated with a missed diagnosis of stroke (12) and the lack of or a delayed diagnosis can lead to increased acute mortality (13). In particular, previous studies (7, 11–14) have shown that about 10% of patients with cerebellar stroke may at least initially present with symptoms that mimic vestibular neuritis (“pseudovestibular neuritis”). These cases, which may present with a National Institute of Health stroke scale of zero, are not uncommonly misdiagnosed, especially by those without sub-specialty training in neuro-otology such as emergency physicians, internists, or even general neurologists (15, 16).

At least two diagnostic approaches have been proposed for the clinical study of patients with acute vertigo/unsteadiness, HINTS (14), and ABCD_2_ (17, 18). The first, an algorithm performed by neuro-otologists has proven very sensitive (over 95%) and specific in a highly selected (high-stroke risk) population, but may not be generalizable to non-sub-specialists in routine practice. More importantly, the HINTS did not include how to evaluate acutely dizzy patients who did not show spontaneous nystagmus. For this reason, we introduced our algorithm that incorporates positional nystagmus and gait evaluation. The ABCD_2_ is a risk assessment tool for predicting stroke, initially created to stratify patients with transient cerebral ischemia of the anterior circulation (17). In patients complaining of continuous vertigo or dizziness ABCD_2_ has shown lower sensitivity and specificity (roughly 60%) than the HINTS for the diagnosis of stroke (18).

The present study is a planned, prospective evaluation of the reliability, and diagnostic accuracy of a simple clinical algorithm we have previously published (19) for the diagnosis of the cause of acute vertigo in the ED.

## Materials and Methods

### Study Design

Single center, non-profit, prospective accuracy study, registered on clinicaltrial.org as NCT02782962, conducted on consecutive adult patients presenting with vertigo or unsteadiness as defined by the consensus document of the committee for the classification of vestibular disorders of the Bárány Society (1).

### Study Setting

Patients were recruited from October 2015 to March 2016 in the ED of a tertiary teaching hospital in Florence, Italy, with an annual census of 130,000 patients. There was no overlap with prior published study populations. According to the study protocol, written informed consent was obtained from the patients or from their relatives for inclusion in the study. All subjects gave written informed consent in accordance with the Declaration of Helsinki. The protocol was approved by the local ethics committee.

### Study Population

Consecutive adult patients presenting to the ED of the University Hospital Careggi for acute (started within 1 week) vertigo/unsteadiness were screened for the study, 24 h a day, 7 days/week during the study period. The two terms “vertigo” and “unsteadiness,” according to Bárány Society consensus (1), are described as “the sensation of self-motion when no self-motion is occurring” and as “the feeling of being unstable while seated, standing, or walking without a particular directional preference,” respectively. We decided to not include “dizziness” among the complaints leading to the inclusion of patients firstly because a similar concept does not exist in formal Italian medical terminology and also because the Bárány Society consensus warned against using the term “dizziness” that may indicate also “pure sensation of impending faint (pre-syncope), disordered thinking (mental confusion), or detachment from reality (depersonalization or derealization) when such sensation is unaccompanied by a sense of spatial disorientation.” Consequently, patients with pseudovertigo, i.e., orthostatic hypotension, anemia, hypoglycemia, electrolytic disorders, or other medical causes of dizziness, were not considered for the study (19).

Patients unable to participate (those with severe dementia, bedridden patient), unable to be followed for 3 months, with terminal disease (less than three estimated months of survival), with known cervical spine, and neck diseases to whom positioning may be dangerous or who refused to participate the study were excluded.

### Management of Patients

The attending emergency physician was blind to the results of the clinical algorithm results, determined the need for any blood tests, diagnostic imaging, therapy, admission, or period of observation or discharge from the hospital. Patients were managed independently from their inclusion in the study and from STANDING results.

### The STANDING Algorithm

**Figure 1:**
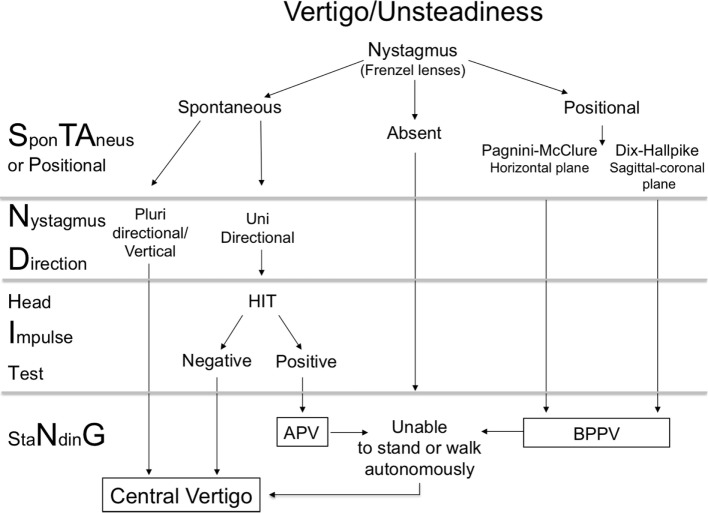
Diagram of STANDING approach. APV, acute peripheral vestibulopathy; BPPV, benign paroxysmal positional vertigo. Central vertigo was diagnosed when an acute brain process (ischemic, hemorrhagic, neoplastic, and inflammatory) congruent with symptoms was detected by neuroimaging or by autopsy (see Materials and Methods).

The STANDING is a structured diagnostic algorithm based on previously described diagnostic signs or bedside maneuvers that we have combined into a four-step sequence (Figure 1) (19).

(1) Assessment of the presence and of type of nystagmus (**S**pon**TA**neous, positional, absent).
(2) Assessment of nystagmus direction (**N**ystagmus **D**irection).
(3) Head **I**mpulse Test (H**I**T).
(4) Evaluation of the standing position and gait (sta**N**din**G**).

(1) The presence of nystagmus is assessed with Frenzel lenses in a supine position after at least 5 min of rest. When no spontaneous nystagmus is detected in primary position and in the five main gaze positions, the presence of a positional nystagmus is assessed by the Pagnini–McClure test (supine roll test) first and then by the Dix–Hallpike test (5). The presence of a positional, paroxysmal (lasting 1–2 min) nystagmus, beating on the plane of the investigated canal (counterclockwise, from the observer’s perspective, and up for posterior right canal, clockwise, from the observer’s perspective, and up for posterior left canal, horizontal for the lateral canals) is considered typical of benign positional paroxysmal vertigo.

(2) When spontaneous nystagmus is present, the direction (from the patient’s perspective) is then examined: multidirectional nystagmus, such as bidirectional gaze-evoked nystagmus (i.e., right beating nystagmus present with rightward gaze and left beating nystagmus present with leftward gaze), or vertical (up or down beating) nystagmus are considered signs of central vertigo.

(3) When there is a spontaneous nystagmus and it is horizontal and unidirectional (i.e., horizontal nystagmus beating on the same side independent of the gaze direction) the HIT is performed (13). When an acute unilateral labyrinthine lesion exists, the input from the opposite side is unopposed resulting in the eyes moving with the head, when the latter is rapidly moved toward the affected side. Immediately thereafter, a corrective eye movement (corrective “saccade”) back to the point of reference is seen. When the corrective “saccade” is present, the HIT is considered positive and it indicates a peripheral disorder, whereas a negative HIT indicates central vertigo (14). These components of the ocular-motor exam have been recently described in detail (20).

(4) At the end of nystagmus examination, all patients, and in particular those showing neither spontaneous nor positional nystagmus, are asked to stand and the gait is evaluated. When objective imbalance is present (inability to stand and walk without assistance) they are suspected to have central vertigo (20).

The real novelties of the STANDING are (a) the sequence (of the tests, i.e., the algorithm), (b) the environment (the ED), and in particular (c) who performed the algorithm: non-sub-specialists in neuro-otology.

One of six specially trained emergency physicians performed the test. The initial training consisted of a 6-h workshop, 4 h of lectures, and 2-h demonstration of STANDING on normal volunteers (five STANDING examinations). Moreover, STANDING trained physicians then performed 10 proctored examinations on ED patients and 1-month use in daily practice under the supervision of a neuro-otologist (Paolo Vannucchi or Rudi Pecci). During the study period, among all physicians working in our ED, only these six physicians have been instructed about the STANDING, and each of these physicians covered 8 h of the 24 h-duty in ED.

Immediately after the exam was done, the STANDING test results were reported on a dedicated data sheet (see Data Sheet 1 in Supplementary Material) and were not shared with the attending emergency physician. At the same time, patient data were collected anonymously in a prospectively designed database (see Data Sheet 2 in Supplementary Material). The STANDING was performed before any neuroimaging testing. The physician who performed the STANDING was blinded to the patient’s clinical data, except those detectable at the time of the STANDING evaluation.

### Final Diagnosis of Central Vertigo

Due to costs and internal policy, not all patients presenting to the ED for vertigo/unsteadiness were routinely studied with magnetic resonance imaging (MRI). Thus, the authors, together with the local ethics committee, established as a reference standard the performance of neuroimaging tests [computed tomography (CT) or MRI of the head] only in case of clinically suspected central disease. In addition, all patients were followed for 3 months. A complete MRI study including diffusion-weighted imaging (DWI) was performed in all cases when a central cause could not be otherwise excluded. This DWI study was performed at least 24 h after symptoms onset. Discharged patients were instructed to return the ED or to contact the neuro-otology unit in case of worsening, recurrent, or unexpected persistence of symptoms or in case of new neurological symptoms. These conditions triggered more diagnostic testing including more neuroimaging tests, as needed to ascertain the diagnosis. All included patients were followed for 3 months and were re-evaluated by the neuro-otologists (Paolo Vannucchi and Rudi Pecci) within 1 week from the index visit and again after 3 months. The following events were recorded during follow-up: death (due to any cause or due to neurological disease), an objective diagnosis of stroke, demyelinating disease, neoplasm, or other new brain disease.

The final diagnosis was established by a panel of experts consisting of an emergency physician (Stefano Grifoni), a neurologist with expertise in neuroimaging (Marco Moretti), and an experienced neuro-otologist (Rudi Pecci), based on all clinical and instrumental data collected during the 3-month follow-up, except for the STANDING results. All neuroimaging digital imaging data performed during the index visit and follow-up were reassessed. Central vertigo was diagnosed when an acute brain process (ischemic, hemorrhagic, neoplastic, and inflammatory) congruent with symptoms was detected by neuroimaging or by autopsy.

### Statistical Analysis

#### Sample Size

On the basis of preliminary data (19), the estimated prevalence of central disease was 10%. Based on this assumption and considering a drop-out of 10%, the minimal number of patients needed to estimate the sensitivity of the test (STANDING) maintaining the 95% confidence intervals (CIs) within 5%, was 330.

#### Methodology

Continuous variables are reported as mean ± SDs when normally distributed, and as median ± inter-quartile range when not normally distributed. Dichotomous variables are reported as percentages with 95% CI. We evaluated STANDING diagnostic accuracy for diagnosis of central vertigo, as above defined, calculating sensitivity and specificity, positive and negative predictive values, with 95% CIs. The STANDING was considered indicative of central vertigo (i.e., worrisome STANDING) when at least one of the following was present (Figure 1): (1) spontaneous vertical or multidirectional nystagmus, (2) spontaneous unidirectional nystagmus with negative HIT, and (3) the patient was unable to stand and walk autonomously, particularly when no nystagmus was found (neither spontaneous nor positional). Accordingly sensitivity was the proportion of worrisome STANDING in patients with final diagnosis of central vertigo, specificity was the proportion of a benign STANDING in patients without final diagnosis of central vertigo, the positive predictive value was the proportion of worrisome STANDING with a final diagnosis of central vertigo (true positives) in all patients with worrisome STANDING and the negative predictive value was the proportion of benign STANDING without final diagnosis of central vertigo (true negatives) in patients without final diagnosis of central vertigo.

The inter-observer agreement was estimated in the first 120 consecutive patients, comparing the test results performed by 2 of 6 emergency physicians on the same patient, calculating the *K* for whole algorithm (the presence or absence of worrisome STANDING), and for each step of the algorithm, according to the method described by Fleiss et al. (21) in case of multiple raters.

To assess the added diagnostic value of the STANDING algorithm in addition to the classical physical examination of patients with vertigo/imbalance, we used the backward stepwise multivariable logistic regression analysis (22). We included in the regression model all the clinical variables associated with the final diagnosis of central vertigo at a significance level of *P* less than 0.1 (Table 1): final results of STANDING, history of hypertension, the presence of continuous vertigo, of headache, and of at least one neurological sign. The final model retained variables associated with the outcome at a significance level of *P* less than 0.05.

**Table 1:** Clinical characteristics at presentation of included patients.

	Overall, *N* = 352	Central vertigo, *N* = 40	Others, *N* = 312
**Clinical characteristics**			
Age, years (median, IQR)	59 (43–72)	73 (55–79)*	58 (42–70)
Female gender	210 (59.7%)	22 (60.3%)	188 (55%)
**Cardiovascular risk factors**			
Hypertension	117 (33.2%)	24 (60%)*	93 (29.8%)
Diabetes	21 (6%)	2 (5%)	19 (6.1%)
Atrial fibrillation	9 (2.6%)	0 (0%)	9 (2.9%)
Previous TIA/stroke	18 (5%)	2 (5%)	16 (5.1%)
Current cigarette smoking	27 (7.7%)	3 (7.5%)	24 (7.7%)
Dyslipidemia	48 (13.7%)	6 (15%)	42 (13.5%)
**Symptoms of presentation**			
Continuous vertigo	67 (19%)	24 (60%)*	43 (13.8%)
Headache	29 (8.2%)	6 (15%)	23 (7.4%)
Hearing loss	17 (4.8%)	1 (2.5%)	16 (5.1%)
Tinnitus	10 (2.8%)	1 (2.5%)	9 (2.8%)
Vomiting	120 (34.1%)	16 (40%)	104 (33.4%)
**Clinical signs**			
Heart rate (bpm ± SD)	74 ± 12	74 ± 12	74 ± 12
Systolic blood pressure (mmHg ± SD)	141 ± 22	153 ± 21*	140 ± 22
Diastolic blood pressure (mmHg ± SD)	77 ± 13	84 ± 12*	77 ± 13
Cranial nerve dysfunction	11 (2.1%)	10 (25%)*	3 (1%)
Limb weakness	14 (4%)	10 (25%)*	4 (1.3%)
Dysarthria/dysphagia	7 (2%)	4 (10%*)	3 (1%)
Limb ataxia	8 (2.3%)	8 (20%*)	0 (0%)
No neurological signs	321 (91.2%)	19 (47.5%*)	302 (96.8%)
**Initial imaging tests^a^**			
Head CT	137 (38.9%)	36 (90%*)	101 (32.4%)
Head MRI	27 (7.7%)	12 (30%*)	15 (4.8%)

*CT, computed tomography; IQR, inter-quartile range; MRI, magnetic resonance imaging; TIA, transient ischemic attack*.

*P < 0.01 vs others.

*^a^Initial imaging test, head imaging during the permanence in the emergency department*.

Dichotomous variables and percentages were compared by χ^2^ test and a two-tailed value of *P* < 0.05 was used to indicate statistical significance. Statistical analysis was performed using SPSS Package 19.0 software (SPSS, Inc., Chicago, IL, USA).

## Results

**Figure 2:**
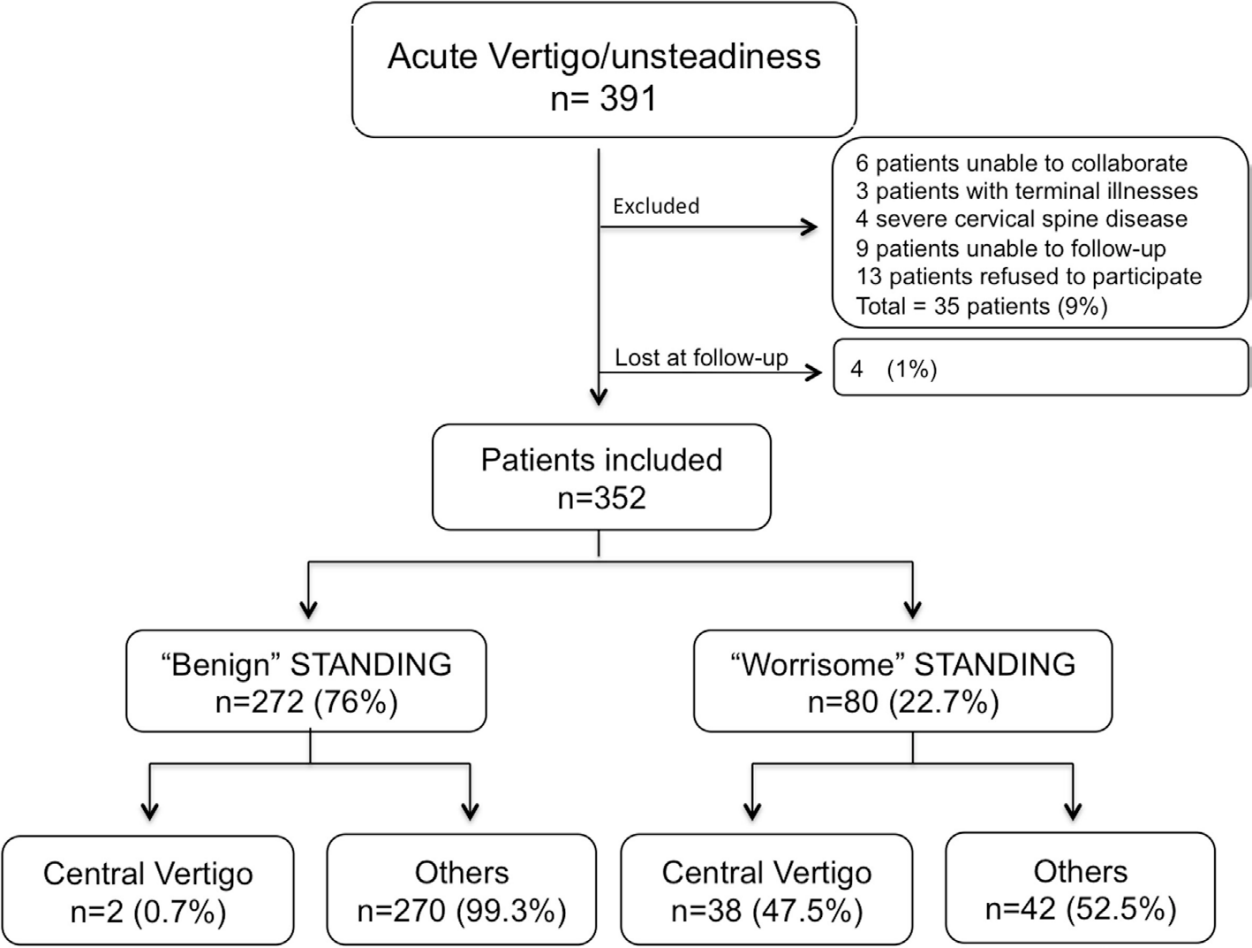
Patient flow diagram.

Among 391 consecutive patients screened for inclusion, 4 (1%) patients were lost at follow-up, and 352 (90%) patients were included in the study (Figure 2).

### General Characteristics of Subject Investigated

Included patients had a mean age of 58 ± 18 years (range 18–99); 59.7% were female (Table 1). Forty (11.4%) patients had a final diagnosis of central vertigo. We diagnosed central vertigo by initial head CT in 12 (30%) patients (7 tumors, 3 ischemic stroke, 1 hemorrhagic stroke, and 1 hydrocephalus), by repeat head CT within 48 h with or without contrast in 8 (20%), and by MRI in 20 (50%) patients. Patients with a final diagnosis of central vertigo were older than those without (*P* < 0.001) (Table 1). The prevalence of arterial hypertension together with systolic and diastolic blood pressure values measured at presentation was higher in patients with a final diagnosis of central vertigo (*P* < 0.01 for all). Continuous vertigo (i.e., constant vertigo lasting hours or days) was more often present among patients with central vertigo (60%) compared with those with other diagnoses (13.8%) (*P* < 0.01). Also the presence of an acute vestibular syndrome (25%), characterized by continuous vertigo, nausea/vomiting, and spontaneous nystagmus, was associated with central vertigo (*P* < 0.01) (Table 2). Conversely, the presence of positional nystagmus (53.4%) was inversely related to the presence of central vertigo (*P* < 0.01) (Table 2). The presence of neurological signs was highly predictive of central vertigo, but only about half of the patients with a final diagnosis of central vertigo had associated neurological signs (Table 1).

**Table 2:** Findings revealed by STANDING algorithm.

	Overall, *N* = 352	Central vertigo, *N* = 40	Others, *N* = 312
Positional nystagmus	188 (53.4%)	3 (7.5%)*	185 (59.3%)
Lateral canal	71 (20.2%)	1 (2.5%)*	70 (22.4%)
Posterior canal	117 (33.2%)	2 (5%)*	115 (36.9%)
Spontaneous nystagmus	88 (25%)	18 (45%)*	70 (22%)
Horizontal unidirectional	64 (18.2%)	3 (7.5%)*	61 (19.6%)
Multidirectional-vertical	24 (6%)	15 (37.5%)*	9 (2.9%)
Positive head impulse test	52 (14.8%)	2 (5%)	50 (16%)
Negative head impulse test	12 (3.4%)	3 (7.5%)	9 (2.9%)
No nystagmus	76 (21.5%)	20 (50%)*	56 (17.9%)
Unable to stand or walk autonomously	69 (20.5%)	33 (82.5%)*	36 (11.5%)
Conclusion: central vertigo	80 (22.7%)	38 (95%)*	42 (13.5%)

*P < 0.01 vs others.

Posterior circulation ischemic stroke was the main diagnosis (67.5%) among patients with central vertigo, followed by neoplastic disease (25%) (Table 3). Benign paroxysmal positional vertigo (56.4%) represented the main diagnosis of the other group, followed by acute peripheral vestibulopathy (17.9%). This latter term includes patients with vestibular neuritis and labyrinthitis.

**Table 3:** Main diagnoses of included patients.

**Central vertigo**	
Ischemic stroke	27 (7.6%)
Hemorrhagic stroke	1 (0.3%)
Cerebral tumors	10 (2.8%)
Hydrocephalus	1 (0.3%)
Demyelinating disease	1 (0.3%)
**Other diagnoses**	
Benign positional paroxysmal vertigo	176 (50%)
Acute peripheral vestibulopathy^a^	56 (15.9%)
Migraine	19 (5.4%)
Vertebro-basilar insufficiency^b^	19 (5.4%)
Meniere’s disease	4 (1.1%)
Miscellaneous	14 (4%)
Undetermined	24 (6.8%)

*^a^Acute peripheral vestibulopathy included both vestibular neuritis and labyrinthitis. Miscellaneous: included toxic or traumatic injury of the inner ear, pseudovertigo due to hyperventilation in anxiety disorders, superior semicircular canal dehiscence*.

*^b^Vertebro-basilar insufficiency was diagnosed when an ischemic etiology was clinically suspected but no new acute lesions were found at neuroimaging. As stated in Section “Materials and Methods,” we included in the central vertigo group only patients with an acute cerebral process detected by head imaging*.

Nineteen patients with central vertigo did not show any additional neurological sign/symptom at the time of physical examination, including headache. Twelve (63%) patients had a final diagnosis of stroke, 10 in the vascular territory of the posterior inferior cerebellar artery (8 in the right or left cerebellar hemispheres and 2 including also the nodulus), 1 in the territory of the anterior inferior cerebellar artery, and 1 showed the dissection of the right vertebral artery without clear ischemia on the MRI. Of the other seven non-ischemic patients, one had hydrocephalus, two had demyelinating disease, two had a primary cerebral neoplasm (one of the right cerebellar hemisphere and one of the acoustic nerve, both presenting with positional nystagmus), and two patients had metastatic cerebral neoplasms (one with multiple secondary lesions, the main cerebellar, from colon cancer and one with a final diagnoses of meningeal lymphoma in a patients with an already known non-Hodgkin lymphoma).

During the 3-month follow-up two patients died, one due to cerebral metastasis and the other from basilar artery obstruction.

### Reliability and Accuracy of STANDING Diagnostic Algorithm

Test characteristics of STANDING algorithm are reported in Table 4.

**Table 4:** Diagnostic characteristics of STANDING algorithm for identification of central vertigo by clinical observation of nystagmus and equilibrium.

	Sensitivity % (95% CI)	Specificity % (95% CI)	Positive predictive value % (95% CI)	Negative predictive value % (95% CI)	*K*^a^
Spontaneous vs positional	45 (30–60)	77 (75–79)	20 (14–27)	92 (89–94)	0.83 (78–88)
Multidirectional or vertical	38 (26–48)	97 (96–98)	63 (43–79)	92 (91–94)	0.95 (90–100)
Negative head impulse test	95 (83–99)	18 (16–19)	11 (9–15)	97 (88–99)	0.74 (66–82)
Unable to stand or walk	83 (68–92)	89 (87–90)	48 (40–53)	97 (95–99)	0.81 (75–87)
STANDING	95 (83–99)	87 (85–87)	48 (41–50)	99 (98–100)	0.83 (74–86)

*^a^*K* was calculated on the first 129 out of 352 consecutive patients according to Fleiss et al. (21)*.

Inter-rater reliability of the STANDING was tested on the first 129 consecutive patients. Each step showed excellent agreement, the third step, the HIT, showing the lowest (0.74) and the second step, the evaluation of the direction of the nystagmus, showing the highest (0.95) agreement. The agreement of the final result of the sequential algorithm was very good (0.83).

When we looked at the diagnostic accuracy of each single step, we found that the presence of and the direction of spontaneous nystagmus showed a low sensitivity for the final diagnosis of central vertigo (45 and 38%, respectively). Conversely, negative HIT and an altered equilibrium were the most sensitive findings for central vertigo. In terms of specificity, the best predictors were the presence of a bidirectional or vertical nystagmus and the inability to stand and walk autonomously.

From the diagnostic point of view, a worrisome STANDING result (i.e., the presence of at least one central finding) showed a very good overall accuracy (88%), specificity (87%), and sensitivity (95%). The derived negative (99%) and positive (48%) predictive values of the algorithm were at the top of the range of each single step thus indicating that the final result of the multistep algorithm allowed a better balance between sensitivity and specificity in respect to each single step. In particular, the very high-negative predictive value allowed us to exclude the presence of central vertigo with a high level of certainty.

As a sensitivity analysis, we calculated the diagnostic characteristics of STANDING considering the 19 patients with posterior circulation transient ischemic attack in the central vertigo group. In this case, the overall sensitivity, specificity, and NPV of STANDING were 83% (95% CI 72–91%), 87% (95% CI 85–89%), and 96% (95% CI 94–98%), respectively.

### Added Diagnostic Value of STANDING

In order to assess the diagnostic power of the STANDING algorithm in clinical context, that is, the capability of STANDING to increase the diagnostic probability of a central “origin” of vertigo/unsteadiness in patients with an otherwise normal clinical examination, we compared the diagnostic accuracy obtained by ordinary physical examination with and without STANDING algorithm. The logistic regression analysis showed that beyond the typical clinical variables used to estimate the risk of central vertigo (i.e., the presence of cardiovascular risk factors, continuous vertigo, headache, and of at least one associated neurological sign), a worrisome STANDING result maintained a significant, independent association with the final diagnosis of central vertigo (OR 122, 95% CI 15–943).

## Discussion

This is the first published prospective validation study of a clinical algorithm for the diagnosis of central “origin” of vertigo/unsteadiness. The STANDING algorithm showed good reliability and very high accuracy in excluding dangerous diseases in the hands of emergency physicians.

Our study is the first one that has investigated the reliability of nystagmus evaluation by non-sub-specialized physicians (emergency physicians). We found a good agreement in properly trained emergency physicians, even higher than that reported for clinical elements of the National Institute of Health Stroke Scale (23). These data indicate that nystagmus evaluation is both feasible and reproducible in the emergency setting by non-sub-specialized physicians. If emergency physicians can confidently diagnose patients with vertigo/imbalance, fewer resources (for expensive and time-consuming imaging and consultation) would be required with equivalent or better outcomes. Although our training program (6 h workshop, 10 proctored examinations, 1-month use in daily practice) is sufficient, we have not investigated if a shorter duration of training is enough. That said, there is evidence that emergency physicians usually do not test for nystagmus, so some training is clearly needed (24).

In emergency physicians’ hands, the STANDING algorithm showed high sensitivity (95%) and good specificity (87%) for central vertigo, reaching very high-negative predictive value (99%). Better results were published when the evaluation was done by neuro-otologists (14). Their algorithm called HINTS, added the test of skew but without initial differentiation between spontaneous and positional nystagmus and of gait evaluation, showed a 100% sensitivity and 96% specificity. However, they studied a selected population of high-risk patients with acute vestibular syndrome, with an extremely high prevalence of stroke (76%) as compared findings by us and others (4, 8, 25). In our larger cohort of “unselected” patients, we found two false-negative cases, both with “pseudo-benign” positional vertigo, one with a tumor of the ponto-cerebellar angle, and one with a demyelinating disease. Based on these data, our clinical algorithm appears to be a feasible, standalone test for excluding dangerous disease in everyday clinical practice. Another recent study of unselected patients with an acute vestibular syndrome (37% with stroke) also found that ataxia was useful to distinguish patients with central causes (26).

When looking at improving the clinical criteria to identify vertigo patients who should be undergo urgent neuroimaging test, we found that our algorithm alone, based on a PPV of 48%, could allow reaching a “number needed to image” of one positive every two to three neuroimaging tests. Previous studies reported very high-neuroimaging study rate among patients with vertigo (27), with diagnostic yield for MRI around 20% (28). This suggests that the STANDING algorithm could significantly reduce unnecessary neuroimaging tests.

Finally, our data showed that 7.6% of patients complaining vertigo or unsteadiness in our cohort have an acute stroke. This is higher than that reported in previous cohort studies in ED (4, 8, 25), and could be due to the fact that our hospital is the referral center for audio-vestibular diseases of a large area of Tuscany. Moreover, it is noteworthy that among patients with central nervous system disease, about 50% initially presented without any associated neurological signs, a finding noted by others in different populations (14, 29, 30). These findings highlight that a careful examination for nystagmus is extremely important for early diagnosis of posterior circulation stroke.

Our study has several limitations. First, not all patients had head CT or MRI (no brain imaging in 211 patients, 59.9%). In high-risk populations (acute vestibular syndrome plus cardiovascular risk factors) about 10% of patients with ischemic stroke may present with vestibular “pseudoneuronitis” (14, 29), i.e., an acute vestibular syndrome due to a small vertebro-basilar infarct that resembles a vestibular neuronitis for the presence of a spontaneous nystagmus and an abnormal HIT test. As showed by Kattah et al., adding to the HIT the observation of a gaze-evoked nystagmus (in our algorithm multidirectional nystagmus) reduces this possibility to 2 out of 69 patients with stroke (2.8%). Projecting the same results in our cohort, we can estimate that, considering patients with an acute vestibular syndrome (88, 25%) and among these those at high risk (at least one cardiovascular risk factor, 42), we may have lost roughly 3% of “peudoneuronitis,” that is, one stroke. We tried to limit this potential bias (which could decrease the proportion of patients with central diseases) by a strict 3-month follow-up in the hand of expert neuro-otologists. Nevertheless, about 7% of patients remained with an undetermined diagnosis. Some of these patients may have had central disease and the sensitivity of the STANDING algorithm could have been overestimated. However, if also in this group of patients the incidence of central diseases was the same as in all the included population (11%), only two to three more patients may have been misdiagnosed, thus potentially reducing diagnostic sensitivity and NPV of STANDING to 88 and 98%, respectively. Furthermore, the monocentric nature of the study precludes the estimation of the validity of our clinical algorithm in different environments. In our center, consultation with expert neuro-otologists is always available, thus allowing a rapid improvement of the expertise in vertigo assessment, a resource that is likely not available in other environments (6, 31, 32). A multicenter validation is necessary for reliably applying the STANDING algorithm in clinical practice.

In conclusion, in a large cohort of patients presenting with vertigo or unsteadiness to a single tertiary hospital, STANDING showed good reliability and accuracy, allowing non-sub-specialized physicians to exclude threatening cerebral disease, including stroke, with a high level of certainty. If these data are confirmed by other studies, our algorithm could be the basis of a new clinical approach to patients with vertigo or unsteadiness.

## Ethics Statement

This study was carried out in accordance with the recommendations of STARD guidelines for reporting diagnostic accuracy studies, with written informed consent from all subjects. All subjects gave written informed consent in accordance with the Declaration of Helsinki. The protocol was approved by the Ethics Committee of the Tuscany Sanitary Regional System, Italy.

## Author Contributions

SV, RP, PN, and PV: study concept and design. SV, PN, RP, RS, GP, MM, AP, ClaudiaC, CosimoC, and SB: acquisition of data; analysis and interpretation of data; and statistical analysis. SV, PV, RP, and AP: drafting of the manuscript. JE, PN, GP, and SG: critical revision of the manuscript for important intellectual content. SV, RP, GP, PV, and SG: study supervision.

## Conflict of Interest Statement

The authors declare that the research was conducted in the absence of any commercial or financial relationships that could be construed as a potential conflict of interest.

## Abbreviations

APV, acute peripheral vestibulopathy; BPPV, benign positional paroxysmal vertigo; CI, confidence interval; CT, computed tomography; DWI, diffusion-weighted imaging; ED, emergency department; HIT, head impulse test; HR, heart rate; IQR, inter-quartile range; MRI, magnetic resonance imaging; STANDING, SponTAneus Nystagmus, Direction, head Impulse test, staNdinG; SBP, systolic blood pressure; VBI, vertebro-basilar insufficiency.
