# Supplementary material for: Differential Diagnosis of Vertigo in the Emergency Department: A Prospective Validation Study of the STANDING Algorithm
Source: Front Neurol. 2017 Nov 7;8:590. doi: 10.3389/fneur.2017.00590 (PMC5682038; doi:10.3389/fneur.2017.00590)
Supplement: Supplementary file 1 [file data_sheet_1.docx]

**Patient number………………. Date…………..**

**HR…………….. SAT02………… RR……..**

**SAP……. DAP………… CT……..**

**Past History**

Hypertension………. Diabetes……….. Atrial Fibrillation………….

Prev Stroke/TIA…… Prev Vertigo……….. Diagnosis of prev Vertigo……….

Smoking…….. Dislipidemia……… Other………….

**Time of onset of vertigo/unsteadiness ………………..**

**Continuous Vertigo or positional ……………….**

**Associated symptoms**

Earache…………… Hearing loss……………….. Tinnitus……………….

Vomiting……………. Diaphoresis…………… Diplopia………………

Visual deficit………

**Associated signs**

Cranial nerv dysf……. Limb weakness…….. Dysarthria/ Dysphagia…….

Limb Ataxia……….. Others………………

**Associated events**

Trauma…………. Infections……………….. Toxic……………….

**Therapy:………………………………………………………………………………………….**

**Imaging:**

Eco color Doppler CV……………………………….

Head CT………………………

Head RMI ……………………………………………….

Others………………………………………………………….

**Disposition from ED**

Discharge……. Observation……….. Admitted………………

**Intrahospital follow-up**

Type of ward…….. Days…………

Stroke………….. Ward upgrade…… Neurosurgery……………..

Fibrinolysis……………… Death……………… Cause of death………….

Event Date…………..

**Therapy (inhospital and discharge)**

**………………………………………………………………………………………………………………..**

**3 months Follow-up**

**one week**

new onset symptoms……………………………

new imaging:

Head CT…………………………….

Head MRI…………………………………….. Others……………………

Stroke/TIA…………. New adimission (why)……………….. Type of ward…………

Neurosurgery……… Fibronolysis……………………….. death………………………

Case of death………

**Therapy during follow-up (if different)**

**………………………………………………………………………………………………………………..**
