# Supplementary material for: Differential Diagnosis of Vertigo in the Emergency Department: A Prospective Validation Study of the STANDING Algorithm
Source: Front Neurol. 2017 Nov 7;8:590. doi: 10.3389/fneur.2017.00590 (PMC5682038; doi:10.3389/fneur.2017.00590)
Supplement: Supplementary file 2 [file data_sheet_2.docx]

**Date………….. Age……………..**

**Patient number………………. Weight (kg)……. Height (cm)…………**

**INCLUSION**

Has the patient acute vertigo/unbalance Yes No

Is the patients adult? Yes No

**ESCLUSION**

Does the patient give its consent for study purpose? Yes No

Has the patient severe symptoms that prevent cooperation? Yes No

Has the patient severe cervical disease that prevent diagnostic manoeuvres? Yes No

Has the patient terminal illness (less than three month of estimated survival)? Yes No

Is the patient available for 3 months follow-up? Yes No

**STANDING**

1. **FIRST STEP Presence of nystagmus:**

**a) Spontaneous:**  **Yes No**

**b) Positional:** **Yes No**

Please specify Pagnini dx Yes No (geotropo apogeo)

Pagnini sin Yes No (geotropo apogeo)

Dix Hallpike dx Yes No

Dix Hallpike sin Yes No

**c) Neither spontaneous nor positional: Yes No**

1. **SECOND STEP**
2. **Unidirectional b) Bidirectional c) Vertical**

1. **THIRD STEP**

**Head Impulse test**

1. **Positive** on the right on the left
2. **Negative**
3. **FOURT STEP**

**STANDING:** normal abnormal (ataxia, please describe)

**CONCLUSION of Study MD**

Central AV Peripheral AV

Dott………………………………
